# Yoga dataset: A resource for computer vision-based analysis of Yoga asanas

**DOI:** 10.1016/j.dib.2023.109257

**Published:** 2023-05-23

**Authors:** Yogesh Suryawanshi, Namrata Gunjal, Burhanuddin Kanorewala, Kailas Patil

**Affiliations:** a Vishwakarma University, Pune, India; b Abbott Point of Care, Ottawa, Canada

**Keywords:** Asana, Exercise, Sports, Artificial intelligence, Machine learning, Computer neural network

## Abstract

The practice of yoga has been shown to have numerous benefits for both physical and mental health, and it has gained popularity worldwide as a form of exercise and relaxation. However, yoga postures can be complex and challenging, especially for beginners who may struggle with proper alignment and positioning. To address this issue, there is a need for a dataset of different yoga postures that can be used to develop computer vision algorithms capable of recognizing and analyzing yoga poses. For this we created the image and video datasets of different yoga asana using the mobile device Samsung Galaxy M30s. The dataset contains images and videos of effective (right) and ineffective postures for 10 Yoga asana, with a total of 11,344 images and 80 videos. The image dataset is organized into 10 subfolders, each with "Effective (right) Steps" and “Ineffective (wrong) Steps" folders. The video dataset has 4 videos for each posture, with 40 videos demonstrating effective (right) postures and 40 demonstrating ineffective (wrong) postures. This dataset benefits app developers, machine learning researchers, Yoga instructors, and practitioners, who can use it to develop apps, train computer vision algorithms, and improve their practice. We strongly believe that this type of dataset would provide the foundation for the development of new technologies that can help individuals improve their Yoga practice, such as posture detection and correction tools or personalized recommendations based on individual abilities and needs.

**Specifications Table**

**Table:** 

Subject	Health, Data Science, Applied Machine Learning

Specific subject area	Image and Video Datasets of Anantasana, Ardhakati Chakrasana, Bhujangasana, Kati Chakrasana, Marjariasana, Parvatasana, Sarvangasana, Tadasana, Vajrasana, and Viparita Karani Yoga asana.
Type of data	Image Video
How the data were acquired	The mobile phone camera was used to capture high-quality images and videos of both correct (right) and incorrect (wrong) Yoga postures, under various backgrounds and lighting conditions.
Data format	Raw
Description of data collection	The image dataset of various effective (right) and ineffective (wrong) Yoga images were captured by mobile camera model Samsung Galaxy M30s. Similarly the video dataset of different effective (right) and ineffective (wrong) Yoga videos were recorded on the same device with 1280 × 720 resolution. The images were captured at resolution with 3264 × 1504 pixel. The images that were taken were saved in ten separate folders, which were named after Yoga postures such as Anantasana, Ardhakati Chakrasana, Bhujangasana, Kati Chakrasana, Marjariasana, Parvatasana, Sarvangasana, Tadasana, Vajrasana, and Viparita Karani. The Yoga posture images were further divided into two categories, namely "Effective" and "Ineffective" steps. Each of these categories had separate folders that contained stepwise images.Effective Yoga asana posture images are stored in the folder named as "Right" and Ineffective Yoga asana posture images are stored in the folder name "Wrong". The videos were stored in ten separate folders, which were also subdivided into Effective and Ineffective categories. The effective category videos are stored in the folder named as "Right" and Ineffective category videos are stored in the folder named as "Wrong" . Each of these categories contained four different angles of both correct and incorrect postures. During the video recording, information about the correct and different incorrect postures was recorded in English language.
Data source location	The dataset presented in this article is prepared at Vishwakarma University, Pune, Maharashtra, India. Latitude and longitude: 18.4603°N, 73.8836°E
Data accessibility	1. For original size images videos: Repository name: Zenodo Data identification number: 10.5281/zenodo.7818789 Direct URL to data: https://doi.org/10.5281/zenodo.7818789 2. For compressed images and videos: Repository name: Mendeley Data Data identification number: 10.17632/jc4mmnvcdk.1 Direct URL to data: https://data.mendeley.com/datasets/jc4mmnvcdk/1

## Value of the Data

• Yoga image and video dataset has significant value for individuals and organizations interested in studying and improving Yoga practice.
• This dataset provides a collection of high-quality images and videos of correct and incorrect Yoga postures captured under different backgrounds and lighting conditions.
• The dataset contains 11,344 number of images and 80 number of videos of various stepwise correct (right) and incorrect (wrong) Yoga postures.
• This dataset is useful in the field of biomechanics, physiology, and sports sciences. It is also useful to analyze the posture of individuals in different poses, assess the impact of different postures on the body, and investigate the benefits Yoga practice.
• This dataset is beneficial for app developers, machine learning researchers, Yoga practitioners and Yoga instructors.
• Machine learning researchers can use this dataset to train computer vision algorithms to recognize and classify different Yoga postures automatically and the app developers can use the dataset to create Yoga apps that provide users with visual guidance on how to perform each pose and track their progress.

## 1 Objective

The main objective to crate this dataset is to develop fitness and wellness applications that offer personalized Yoga instruction and feedback based on real-time analysis of the user's posture. By studying images and videos of correct posture and technique, practitioners can learn how to adjust their own posture and movements to optimize their practice. Additionally, this dataset could be used for research purposes, such as developing automatic posture recognition models to improve Yoga practice.

## 2 Data Description

The term "Yoga asana" refers to the various physical postures or positions that are performed in the practice of Yoga, with the aim of enhancing flexibility, strength, balance, and relaxation [1]. These postures are typically held for a period of time and are often accompanied by controlled breathing and meditation, and offer numerous physical and mental benefits [2,3].

The dataset comprises both images and videos depicting right and wrong postures for a variety of Yoga asanas. The focus of the dataset is on 10 specific Yoga postures, namely Anantasana (Sleeping Vishnu Pose, Vishnu's Couch Pose, Eternal One's Pose, or Side-Reclining Leg Lift); Ardhakati Chakrasana (Lateral Arc Pose); Bhujangasana (Cobra Pose, Sphinx Pose, Serpent Pose); Kati Chakrasana (Standing Spinal Twist Pose); Marjariasana (Bidalasana, Cat Pose); Parvatasana (Mountain Pose); Sarvangasana (Shoulder Stand Pose, Salamba Sarvangasana); Tadasana (Samasthiti, Standing Mountain Pose); Vajrasana (Thunderbolt Pose, or Diamond Pose); and Viparita Karani (Legs Up The Wall Pose). Table 1 shows some sample images of different effective (right) and ineffective (wrong) Yoga asana postures.

**Table 1:** Sample images of different Yoga asana postures.

Name of Yoga asana	Effective posture	Ineffective posture	Details about ineffective postures
Anantasana			Wrong Leg bending, Hand and Neck position
Ardhakati Chakrasana			Wrong Legs distance and hand position
Bhujangasana			Wrong Legs distance and hand position
Kati Chakrasana			Wrong Neck and hand position
Marjariasana			Wrong leg distance, hand bending
Parvatasana			Wrong hand position
Sarvangasana			Wrong back, legs and hand position
Tadasana			Wrong hand bending and leg distance
Vajrasana			Wrong back and neck position
Viparita Karani			Wrong leg bending and hand position

The Image dataset comprises a total of 11,344 images and is organized into 10 subfolders, each corresponding to a specific Yoga asana. Within each subfolder, there are two additional folders labelled "Right Steps" and "Wrong Steps". The "Right Steps" folder contains several subfolders, each representing a specific step in the correct sequence of the Yoga asana and displaying the corresponding images. On the other hand, the "Wrong Steps" folder includes multiple subfolders, each showing images of a incorrect steps in the sequence of the Yoga asana. The details of Yoga asana image numbers are presented in Table 2.

**Table 2:** Details of Yoga asana image dataset.

Asana name	Correct posture photos	Incorrect posture photos	Total photos
Anantasana	640	535	1175
Ardhakati Chakrasana	507	420	927
Bhujangasana	684	479	1163
Kati Chakrasana	320	225	545
Marjariasana	845	748	1593
Parvatasana	1375	1402	2777
Sarvangasana	347	359	706
Tadasana	854	718	1572
Vajrasana	65	86	151
Viparita Karani	376	359	735
**Total**	6013	5331	11,344

The Yoga asana video dataset consists of 8 videos for each posture, comprising 4 videos demonstrating the effective posture from 4 different angles and 4 videos exhibiting the ineffective posture from 4 different angles. This dataset includes a total of 80 videos for 10 Yoga asanas, with 40 videos demonstrating the effective postures captured from 4 different angles, and 40 videos illustrating the ineffective postures from 4 different angles. The details of Yoga asana video numbers are presented in Table 3.

**Table 3:** Details of Yoga asana video dataset.

Asana name	Correct posture video	Incorrect posture video	Total Video
Anantasana	4	4	8
Ardhakati Chakrasana	4	4	8
Bhujangasana	4	4	8
Kati Chakrasana	4	4	8
Marjariasana	4	4	8
Parvatasana	4	4	8
Sarvangasana	4	4	8
Tadasana	4	4	8
Vajrasana	4	4	8
Viparita Karani	4	4	8
**Total**	40	40	80

The Yoga asana image dataset is important for improving Yoga practice, enhancing teaching, supporting research, advancing technology, and promoting accessibility. Previous research works have created Yoga datasets, however those datasets do not cover effective and ineffective ways of doing yoga asana [13,14]. Moreover, these datasets represents various yoga poses but not the complete Yoga asana steps from start to end.

Therefore these datasets have limitations in Artificial Intelligence or Machine learning based applications to detect ineffective ways of doing asana. Furthermore, those datasets contain either images or videos. To address these limitations of existing datasets we created this dataset that contains various images and videos of incorrect postures in Yoga asanas which is important to help in identifying and correcting common mistakes made during the practice of Yoga. The dataset provides a visual reference for the correct alignment and posture in each yoga pose, allowing practitioners to improve their practice and avoid injury. Yoga instructors can use the dataset to demonstrate correct postures and alignment, which can enhance the quality of instruction and help students achieve optimal results. By studying the incorrect posture images, practitioners and instructors can better understand the mistakes that are commonly made and can work to correct them. The dataset can be used by researchers to investigate the effects of Yoga on the body and to develop new techniques and practices to improve overall well-being [4,5]. Additionally, this dataset can help in the development of machine learning models that can automatically identify and correct improper postures during Yoga practice [[6], [7], [8]]. Machine learning can help to identify various things, including but not limited to, images, patterns, objects, text, and anomalies in data [9].

## 3 Experimental Design, Materials and Methods

### 3.1 Experimental Design

The high-definition rear camera of mobilephone was used to capture the photographs and videos of different Yoga asanas postures. All the photographs and videos were taken with a mobile same camera and separated into different folders based on their type of Yoga asana and "Right" and "Wrong" steps. During February 2023 to March 2023 pictures and videos of different Yoga asana posture image and videos captured in natural and artificial light, from various angles, and different background. This type of diverse dataset of Yoga asana images and videos captured in different lighting conditions and angles can benefit computer vision research, improve user experience in Yoga applications, enhance research on the impact of Yoga on health, and increase accessibility for people with different abilities and body types.

### 3.2 Experimental Material

The dataset was created using a Samsung Galaxy M30s Mobile with a 48 megapixel camera for image capturing and video recording. Multiple photos and videos were taken for each Yoga asana, including effective and ineffective postures. Images were saved in JPG format, while videos were saved in MP4 format. The images were captured in different outfits, environmental settings, lighting conditions, backgrounds, and from various angles. Videos were recorded from four different angles, which included front, left side, right side, and back side, and featured instructions in English.

### 3.3 Experimental Method

#### 3.3.1 Posture Categorisation

The Yoga Dataset consist of folders labelled as "Right" and "Wrong." One of the author of the datasets is assistant professor at the department of Yoga and Naturopathy, Vishwakarma University, Pune. He provided guidance and instructions to yoga performers, ensuring they performed the correct and effective yoga poses and postures. The criteria for determining the "Right" postures were based on the book titled "Daily Yoga Practice Routine" [10]. The "Wrong" postures were intentionally created to depict common postures practised by individuals with limited knowledge of yoga and pranayama, which may be ineffective or incorrect. This dataset are created based on ancient Indian literature, ensuring their accuracy, reusability, reproducibility, and value to various research communities [15].

#### 3.3.2 Procedure for Constructing a Dataset

**Fig. 1:**
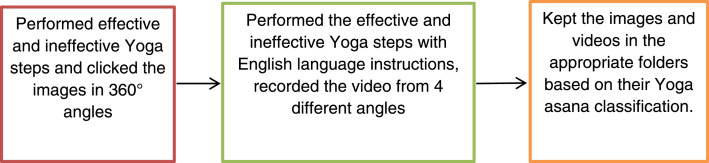
Yoga asana data acquisition process.

The images were captured and videos recorded at various environmental locations. The effective Yoga asana postures are performed as described in the book Daily Yogapractice Routine [10]. The ineffective postures were intentionally created to depict common postures practised by individuals with limited knowledge of Yoga asana, which may be ineffective or incorrect. Fig. 1 displays the different Yoga asana image and video data acquisition procedure. The images were captured in diffeent angles of effective and ineffective postures. The Samsung Galaxy M30s smartphone camera, which has an F-stop of f/2.2, exposure time of 1/20 sec, ISO speed of 500, focal length of 1 mm, no flash mode and centre weighted average settings were used to capture the images of the dataset. The images have a dimension of 3264 × 1504 pixels, with a width of 3264 pixels and a height of 1504 pixels. The horizontal and vertical resolutions are both 72 dpi. The images have a bit depth of 24 and use the sRGB colour representation. The images are of the JPG file type. These details are important for understanding the size, resolution, and format of the acquired images. The captured images were systematically organized and saved in ten distinct folders, each named after a specific Yoga posture. These included Anantasana, Ardhakati Chakrasana, Bhujangasana, Kati Chakrasana, Marjariasana, Parvatasana, Sarvangasana, Tadasana, Vajrasana, and Viparita Karani. Moreover, each Yoga posture had two sub-folders, "Right" and "Wrong" steps, with step-by-step images of both the correct and incorrect postures.

The videos were recorded with same mobile device. The videos have a frame width of 1280 pixels and a frame height of 720 pixels. The frame rate of the videos is 30.01 frames per second. The audio bit rate ranges from 255-257 kbps, and the audio sample rate is 48.000 kHz. The videos are recorded in MP4 format. The videos were categorized into ten separate folders, further divided into "Right" and "Wrong" categories. These categories contained four distinct angles of both the correct and incorrect postures, recorded in English language with detailed information about the proper and improper techniques. The created dataset is uploded on Zenodo in original raw format and it is publicy available to dowload [11]. The images are compressed using IrfanView software and videos are compressed using VLC software. The compresed dataset of yoga images and videos is available on Mendeley Data [12]. This type of dataset can be used to train machine learning models and identify the correct and incorrect Yoga postures automatically.

## Ethics Statements

The person present in dataset images and videos is one of the author of this dataset. He provided written informed consent to being included in the study and allowing their data to be shared publicly. We confirm that we don't have any conflict of interest.

The research presented in this paper did not conduct the animal or human studies. No ethical approval was necessary for this research, and no living organism was subjected to any harm.

## CRediT authorship contribution statement

**Yogesh Suryawanshi:** Conceptualization, Methodology, Data curation, Writing – original draft. **Namrata Gunjal:** Conceptualization, Writing – review & editing. **Burhanuddin Kanorewala:** Data curation, Methodology. **Kailas Patil:** Supervision, Validation, Writing – review & editing.

## Declaration of Competing Interest

The authors declare that they have no known competing financial interests or personal relationships that could have appeared to influence the work reported in this paper.

## Data Availability

Yoga for all: A Comprehensive Collection of Yoga Images and Videos dataset (Original data) (Mendeley Data).
